# Human SP-D Acts as an Innate Immune Surveillance Molecule Against Androgen-Responsive and Androgen-Resistant Prostate Cancer Cells

**DOI:** 10.3389/fonc.2019.00565

**Published:** 2019-07-11

**Authors:** Gargi Thakur, Gagan Prakash, Vedang Murthy, Nilesh Sable, Santosh Menon, Salman H. Alrokayan, Haseeb A. Khan, Valarmathy Murugaiah, Ganesh Bakshi, Uday Kishore, Taruna Madan

**Affiliations:** ^1^Department of Innate Immunity, ICMR-National Institute for Research in Reproductive Health, Mumbai, India; ^2^Tata Memorial Hospital, Homi Bhabha National Institute, Mumbai, India; ^3^Department of Biochemistry, College of Science, King Saud University, Riyadh, Saudi Arabia; ^4^Biosciences, College of Health and Life Sciences, Brunel University London, Uxbridge, United Kingdom

**Keywords:** pattern recognition receptor, prostate tumor explants, LNCaP cells, PC3 cells, viability, apoptosis, p53, pAkt

## Abstract

Surfactant Protein D (SP-D), a pattern recognition innate immune molecule, has been implicated in the immune surveillance against cancer. A recent report showed an association of decreased SP-D expression in human prostate adenocarcinoma with an increased Gleason score and severity. In the present study, the SP-D expression was evaluated in primary prostate epithelial cells (PrEC) and prostate cancer cell lines. LNCaP, an androgen dependent prostate cancer cell line, exhibited significantly lower mRNA and protein levels of SP-D than PrEC and the androgen independent cell lines (PC3 and DU145). A recombinant fragment of human SP-D, rfhSP-D, showed a dose and time dependent binding to prostate cancer cells via its carbohydrate recognition domain. This study, for the first time, provides evidence of significant and specific cell death of tumor cells in rfhSP-D treated explants as well as primary tumor cells isolated from tissue biopsies of metatstatic prostate cancer patients. Viability of PrEC was not altered by rfhSP-D. Treated LNCaP (p53^+/+^) and PC3 (p53 ^−/−^) cells exhibited reduced cell viability in a dose and time dependent manner and were arrested in G2/M and G1/G0 phase of the cell cycle, respectively. rfhSP-D treated LNCaP cells showed a significant upregulation of p53 whereas a significant downregulation of pAkt was observed in both PC3 and LNCaP cell lines. The rfhSP-D-induced apoptosis signaling cascade involved upregulation of Bax:Bcl2 ratio, cytochrome c and cleaved products of caspase 7. The study concludes that rfhSP-D induces apoptosis in prostate tumor explants as well as in androgen dependent and independent prostate cancer cells via p53 and pAkt pathways.

## Introduction

Prostate cancer, an adenocarcinoma of epithelial cell-origin, is the second most frequently diagnosed cancer among men (1). In its early stages, prostate cancer cells rapidly proliferate in an androgen dependent manner, and thus, are treated by androgen deprivation therapy (2). Conventional anti-cancer treatments such as chemotherapy and radiotherapy improve survival, but many patients encounter relapse and metastasis. Following the remittent stage, the cancer progresses to become androgen-independent where most therapeutic strategies fail.

Immunotherapy by stimulating pattern recognition receptors of the innate immune system such as toll-like receptors (TLRs) has shown promise as a preferred adjunct treatment against cancer (3). Imiquimod, a synthetic imidazoquinoline and an agonist that targets TLR-7 and induces the production of pro-inflammatory cytokines including IFN-α, IL-6, and TNF-α, inhibited cancer growth in the mouse prostate via apoptosis induction (4, 5).

Collectins are pattern recognition proteins belonging to the C-type lectin family. They are composed of an N-terminal cysteine-rich region, a triple-helical collagen domain, an α-helical coiled-coil neck region, and C-terminal carbohydrate recognition domain (CRD) (6). Surfactant Protein D (SP-D) is one of the most studied collectins with a vital role in host defense against pathogens and allergens, and modulation of inflammatory response (6). Although SP-D was historically shown to be lung-resident being produced by type II alveolar and Clara cells (7), studies during the last decade have established its extrapulmonary existence in a range of tissues (8). SP-D has also been shown to be expressed in the male reproductive tracts of human and mice (9, 10). Elevated levels of SP-D at inflamed sites in the prostate manifested protection against bacterial infection (11, 12). Kankavi et al. (13) observed differential expression of SP-D in glandular structures of inflamed malignant and non-malignant human prostate tissues. There was a significant correlation between decreased levels of SP-D and increased Gleason score, a grading system based on the histologic pattern of arrangement of carcinoma cells, and tumor volume.

Previously, we reported a novel anti-cancer role of human SP-D and its recombinant fragment (rfhSP-D) comprising 8 Gly-X-Y repeats neck and CRD region, wherein they reduced the viability of a range of human cancer cell lines including eosinophilic leukemia cell line (AML14.3D10) (14). Importantly, survival of peripheral blood mononuclear cells (PBMCs) derived from healthy individuals was found to be unaltered (14). rfhSP-D treated AML14.3D10 cells showed a significant increase in apoptosis with reduced HMGA1 levels and increased levels of activated p53 and caspase 9 (14). SP-D has recently been shown to inhibit the proliferation, migration and invasion of A549 human lung adenocarcinoma cells by binding to N-glycans of epidermal growth factor receptor (EGFR) via its CRD region, and interfering with EGF signaling (15). In UV treated apoptotic Jurkat T cells, SP-D enhanced membrane and nuclear blebbing, suggesting involvement of SP-D in induction of apoptosis (16). Exogenous treatment of SKOV3 cells (an ovarian cancer cell line) with rfhSP-D led to increased caspase 3 cleavage and induction of pro-apoptotic genes Fas and TNF-α (17).

To take the next logical step from the reported anti-cancer role of SP-D in tumorigenic cell lines, we examined anti-prostate cancer role of rfhSP-D using tumor explants and primary cells derived from tissue biopsies of metastatic prostate cancer patients. rfhSP-D induced apoptosis selectively in various prostate cancer cells including the two prostate cancer cell lines (LNCaP and PC3) in a dose- and time- dependent manner. Apoptotic signaling involved upregulation of p53 and downregulation of pAkt. Decreased levels of Bcl2, with a concomitant increase in Bax, cytochrome c and cleavage of caspase 7, confirmed rfhSP-D mediated induction of intrinsic apoptosis.

## Materials and Methods

### Ethics Statement

The study was approved by the Institutional Ethics Committee for Clinical Studies, ICMR- National Institute for Research in Reproductive Health; (Project No.: 260/2014) and Institutional Ethics Committee, Tata Memorial Hospital (Study no: 1467). Tissue biopsy samples were collected from 9 suspected metastatic prostate cancer patients (treatment naïve) undergoing Transrectal Ultrasound guided multiple core needle biopsy, with written informed consent. Chemotherapy was initiated in the patients after confirmation of metastasized prostate cancer. Information regarding the androgen dependency of the prostate cancer in these patients was not available. Average age of the study participants was 67.4 ± 3.97 years with Mean PSA (Prostate- specific antigen) level of 190.04 ± 85.12 ng/ml and Median Gleason score of 8 ± 0.83.

### Explant and Cell Culture

Prostate tissue biopsies were collected in cold Phosphate Buffer saline containing 10% v/v fetal bovine serum (FBS; GIBCO) and 2% v/v Pen-Strep solution (GIBCO) and immediately processed in sterile conditions. Biopsies were cut into 5 mm explants, placed in the scratched areas of 35 mm tissue culture plate (Nunc) and cultured in RPMI 1640 (GIBCO) supplemented with 10% FBS, 1% Pen-Strep solution, Glucose (1 mg/ml) (HiMedia) and 1% Sodium Pyruvate (GIBCO) (complete RPMI) for a week. Primary Cancer Epithelial Cells (PrCEC) isolated from the explants were sub-cultured using 5 mM EDTA (Sigma) in PBS and passaged further. Isolated PrCEC (Passage 2) were examined for Cytokeratin (an epithelial cell marker) via immunofluorescence microscopy (Anti-cytokeratin antibody, Dako, 1:500) and by Real time RT-PCR analysis for CD164 expression, a marker for metastatic epithelial cell cancer (18). Isolated PrCEC were stained for the presence of Alpha-methylacyl-CoA racemase (AMACR) expression, which is upregulated in prostate cancer with high-grade prostatic intraepithelial neoplasia (HGPIN) than in the normal human prostate (19). PrEC, PrCEC and PC3 cells were stained using mouse monoclonal antibodies to AMACR (Novus Biologicals, 1:100) or matched-isotype control IgG for 1 h at 4°C. Phycoerythrin-conjugated rat anti-mouse IgG (Molecular Probes, Eugene, USA) was used as secondary antibody. The gated cell population was determined using PE tagged anti-AMACR. Cells were analyzed via BD FACS Aria III (BD Biosciences, San Jose, California, USA) flow cytometer.

Human prostate cancer cell lines, LNCaP (androgen dependent, p53^+/+^), DU145 (androgen independent p53^+/−^) and PC3 (androgen independent, p53^−/−^) (ATCC, Rockville, MD, USA) were cultured in complete RPMI 1640 medium. Human primary prostate epithelial cells (PrEC; LONZA) were maintained in Prostate epithelial growth medium (PrEGM Bulletkit) supplemented with Triiodothyronine (T3), Transferrin, Bovine Pituitary Extract (BPE), recombinant human Epidermal growth factor (rhEGF), GA-1000, Insulin, Hydrocortisone, Epinephrine, and retinoic acid (LONZA, Cat no. CC-3165). All experiments with Human PrEC were completed within first five passages. Cells were grown at 37°C under 5% v/v CO_2_ until 70–80% confluency was attained.

### rfhSP-D Preparation

The recombinant fragment of human SP-D (rfhSP-D) was expressed in *Escherichia coli* BL21 (λDE3) pLysS (Invitrogen), purified and characterized, as described previously (15). Endotoxin level in the rfhSP-D preparation was determined using the QCL-1000 Limulus amebocyte lysate system (BioWhittaker Inc., USA). The assay was linear over a range of 0.1–1.0 EU/ml (10 EU = 1 ng of endotoxin) and the amount of endotoxin present in the preparations was found to be <4 pg/μg of rfhSP-D.

### Interaction Between FITC Labeled rfhSP-D and Prostate Cells

rfhSP-D was labeled with FITC dye (20) and incubated with prostate epithelial cells and prostate cancer cells at 5, 10, and 20 μg/ml concentration in staining buffer for 15, 30, 45, and 60 min at 4°C in the presence of 2 mM CaCl_2_. Cells were washed to remove unbound rfhSP-D and fixed with 2% PFA for analysis via BD FACS Aria III (BD Biosciences, San Jose, California, USA). Data was analyzed using FCS Express 6 De Nova software. To assess the specificity of the interaction, PC3 cells were incubated with FITC labeled rfhSP-D in the presence of 5 mM CaCl_2_, or 5 mM EDTA, or 5 mM Glucose in PBS, pH 7.4. Staining buffer was used as control for these experiments.

### Cell Viability Assay

Human PrEC (passage no. 3–5), LNCaP, PC3, or PrCEC (passage no. 3–5) cells (5 × 10^3^) were placed in 96-well tissue culture plates (Nunc) and grown overnight. Cells were then starved in cell appropriate serum free media (PrEC and PrCEC for 4 h; LNCaP cells for 12 h; PC3 cells for 18 h) and treated with rfhSP-D (5, 10, and 20 μg/ml) for 24, 48, and 72 h. Cells alone in the culture medium served as an untreated control. After incubation 10 μl MTT [3-(4, 5-dimethylthiazol-2-yl)-2, 5-diphenyltetrazolium bromide] (5 mg/ml stock) was added to each well and incubated at 37°C for 4 h. Formazan crystals were dissolved in acidified iso-propanol and absorbance was read at 570 nm (Beckman Coulter).

### Cell Cycle Analysis

LNCaP or PC3 cells (2 × 10^4^) were plated in 12-well tissue culture plate, starved for 18 h in serum-free RPMI medium serum-free RPMI medium, and then treated with rfhSP-D (20 μg/ml) for 48 h. After incubation, cells were trypsinized, suspended in cold hypotonic solution containing 0.1% sodium citrate, 0.3 μl/mL of NP-40 (Sigma), 2 mg/mL RNaseA (Thermo Fisher Scientific), and 50 μg/mL Propidium Iodide (PI; Sigma) for 20 min, and then analyzed using BD FACS Aria III using BD FACS DIVA software (21).

### Fluorescence Microscopy for Nuclear Morphology

PrEC, LNCaP, or PC3 cells (2 × 10^3^) were grown on coverslips and incubated with rfhSP-D (20 μg/ml) for 48 h to analyze nuclear morphology following induction of apoptosis. Cells were fixed in 2% PFA and permeabilized using 1% v/v Triton X 100 (Sigma). Cells were incubated with Hoechst (1:10,000, Invitrogen) for 20 min in dark. Coverslips were mounted in vector shield (Vector laboratories, UK) and observed under confocal microscope (Zeiss, Germany).

### TUNEL (Terminal Deoxynucleotidyl Transferase dUTP Nick end Labeling) Assay

Prostate tissue biopsies collected from metastatic prostate cancer patients were incubated with rfhSP-D (40 μg/ml) for 48 h in serum free RPMI medium at 37°C under 5% v/v CO_2_. Five micrometer paraffin embedded sections of 10% NBF (neutral-buffered formalin) fixed prostate tissue biopsies were placed on poly L-lysine coated slides. The sections were fixed with chilled acetone followed by washing with PBS. Slides were incubated in TUNEL Mix (Roche Diagnostics), containing terminal deoxynucleotidyl transferase (TdT) and fluorescein labeled nucleotides for an hour in a moist chamber at 37°C. The slides were washed and counterstained with DAPI (4′,6-diamidino-2-phenylindole). TUNEL positive apoptotic cells (green-stained cells) were viewed under confocal microscope (Zeiss, Germany).

### Annexin V Assay

For Annexin-V immunostaining, the manufacturer's protocol of Annexin V-FITC apoptosis detection kit (Calbiochem) was followed with some modifications. PrEC, LNCaP, or PC3 were treated with indicated concentrations of rfhSP-D and harvested at the end of 24 and 48 h. Cells were trypsinized and washed with ice cold PBS to remove culture supernatant, followed by incubation with FITC-tagged Annexin V for 20 min in dark. Subsequently, Annexin V was washed and 1 μl PI was added to stain the DNA. Cells were immediately analyzed via BD FACS Aria III.

### Western Blot

PC3 or LNCaP cells (1 × 10^6^) were plated in a six-well plate and incubated with or without rfhSP-D (20 μg/ml), in serum-free RPMI medium for 12 h and 24 h. The cells were lysed in lysis buffer (50 mM Tris-HCL, pH7.5, 150 mM NaCl, 1% Triton X, 1 mM Sodium orthovanadate, 10 mM β- glycerophosphate, 2 mM EDTA, 10 mM Sodium pyrophosphate) and analyzed by western blotting. Lysate proteins (30 μg) were separated on 15% SDS-PAGE polyacrylamide gel and electrophoretically transferred onto PVDF membranes (Pall Corporation, NY, USA). The blot was probed with primary antibodies raised against human SP-D [a gift from Uffe Holmskov, (13)], Phosphorylated-p53 (Ser 15) (Cell Signaling Technology), pAKT (PathScan® Multiplex Western Cocktail I), pan Akt (ABclonal), phospho-Bad-S155 (ABclonal), Bcl-2 associated death promoter (Bad) (Apoptosis I sampler Kit), Bcl-2-associated X protein (Bax) (Apoptosis I sampler Kit), B-cell lymphoma 2 (Bcl2) (Apoptosis I sampler Kit) or caspase 7 (Cell Signaling Technology), followed by HRP-conjugated secondary antibodies. All western blot images were acquired by Syngene (Chem Genius) and quantified by Syngene Gene Tools.

### Enzyme-Linked Immnosorbent Assay (ELISA) for SP-D and Cytochrome c

Cell lysates and culture supernatants were analyzed for SP-D levels (Duo Set Human SP-D, catlog no. DY1920, R & D Systems) and Cytochrome c (Human Cyt-C, catlog no. E1516Hu, BT Assay) using commercially available ELISA kits. Briefly, for quantification of cytochrome c, PrEC, LNCaP, PC3 cells, or Prostate tissue biopsies collected from metastatic prostate cancer patients were incubated with indicated concentrations of rfhSP-D for 48 h. Cell culture supernatant was analyzed for cytochrome c released by cells. Color development was stopped using 2N H_2_SO_4_ and optical density was determined at 450 nm using a microplate reader (Beckman Coulter).

### Real Time PCR

PrEC, PrCEC, PC3, and LNCaP cells (1 × 10^6^) were plated in a six-well plate and total RNA was isolated using Trizol (Takara) that was further treated with DNase I (Thermo Scientific, Rockford, USA) at 37°C for 30 min to eliminate genomic DNA contamination. One to two microgram of total RNA was reverse transcribed into cDNA using Superscript III first strand synthesis kit (Invitrogen, USA). One microliter of cDNA was used for real time PCR reactions using BioRad CFX96 TouchTM real-time PCR detection system and iQTM SYBR Green Supermix (Bio-Rad, Hercules, CA, USA). 18S was used as housekeeping control. Each qPCR experiment was performed in triplicates and each experiment was repeated 3 times. Primers were designed using NCBI Primer BLAST Software and their annealing temperatures and product sizes are mentioned in Table 1.

**Table 1:** Primer sequences.

**Transcripts**	**Forward primer (5′-3′)**	**Reverse primer (5′-3′)**	**Tm (°C)**	**Product size (in bp)**
SP-D	AGGCTGCTTTCCTGAGCATGAC	CCATTGGTGAAGATCTCCACACAG	57.8	148
CD164	GACTTTAGCGCCCATCTCCA	GCCGTGGAAACGGAACAGAA	68	233
BCL2	GCGTCAACCGGGAGATGTCGCCC	TTTCTTAAACAGCCTGCAGCTTTG	66	208
BAX	AGTGACCCCTGACCTCACTG	GCAGGGGACTGAGATGAACG	68	296
BAD	GAGCTCCGGAGGATGAGTGA	CAAGTTCCGATCCCACCAGG	68	141
18S	GGAGAGGGAGCCTGAGAAAC	CCTCCAATGGATCCTCGTTA	64	174

### *In silico* Analysis of Human SP-D Promoter Region for Androgen Responsive Elements

*In silico* analysis was carried out to predict putative androgen responsive elements (ARE) in promoter regions of human SP-D gene using MatInspector Genomatix v3.4 Software, GmbH, Munchen (Germany). The transcription start site (TSS) for SP-D was determined from the Institute of Bioinformatics and Applied Biotechnology (IBAB) MGEx-Tdb database. Promoter regions −10,000 bp upstream and +1,000 bp downstream from the transcription start site (TSS sites) were submitted for analysis.

### Statistical Analysis

GraphPad PRISM Software version 6.00 (GraphPad Software Inc., San Diego, CA) was used to plot the graphs and analyze the data using one-way ANOVA with Bonferroni corrections for comparison among prostate cells or unpaired two tailed Student's *t*-test for comparing the rfhSP-D treated groups with control. Data is represented as mean ± SD. Values of *p* < 0.05 were considered statistically significant.

## Results

### Prostate Epithelial and Cancer Cells Expressed SP-D

**Figure 1:**
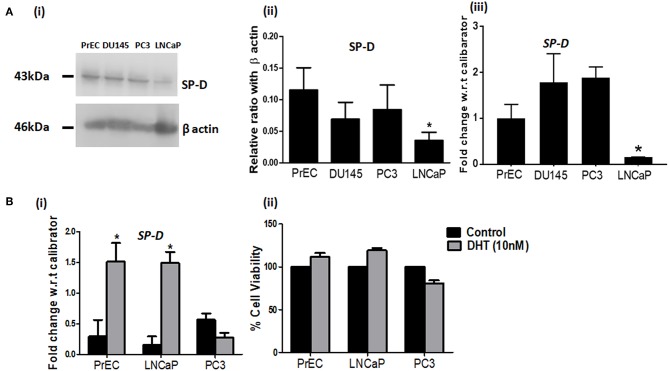
SP-D expression is regulated by Dihydrotestosterone (DHT) in prostate cells. **(A)** Levels of SP-D protein by immunoblotting (i) and transcripts by real time RT-PCR (iii) in PrEC, DU145, PC3, and LNCaP cell lysates. Panel (ii) shows densitometric analysis for the SP-D expression in PrEC, DU145, PC3, and LNCaP cells; *n* = 3 independent experiments, error bar represents the S.D. *p < 0.05. **(B)** (i) SP-D transcripts in PrEC, LNCaP, and PC3 cells treated with DHT (10 nM) for 48 h. *p < 0.05 relative to untreated controls; *n* = 3 independent experiments, error bar represents the S.D. (ii) Cell viability of PrEC, LNCaP, and PC3 cells incubated with DHT (10 nM) for 48 h by MTT assay. Each bar represents % viability ± S.D of three independent experiments. *p < 0.05, relative to untreated controls.

Since it is known that prostate epithelial cells secrete SP-D (11, 13), we evaluated SP-D expression and its regulation in PrEC, LNCaP, DU145, and PC3. Figure 1A shows that SP-D is expressed by prostate epithelial cells and prostate tumor cells; SP-D expression in LNCaP cells is significantly lower in comparison with DU145 (*p* < 0.05), PC3 (*p* < 0.05), and PrEC (*p* < 0.05) by western blot and ELISA (PrEC-1.48 ± 0.31 pg of SP-D/μg of total protein; LNCaP-1.26 ± 0.28 pg/μg; DU145-1.50 ± 0.35 pg/μg, data not shown). SP-D was detectable in the cell culture supernatants of various prostate cells (Range-150 ± 21 to 260 ± 35 pg/ml), suggesting that SP-D is secreted, though not sufficient enough to induce apoptosis (10–20 μg/ml) (14). It is reported that androgen induces proliferation of prostate epithelial cells and LNCaP cells via androgen receptors (22). Furthermore, SP-D expression in rodent prostate is altered on castration (11). Hence, we evaluated levels of SP-D transcripts in Dihydrotestosterone (DHT) treated PrEC, LNCaP, and PC3 cells. Treatment with DHT (10 nM) significantly upregulated SP-D transcripts in both PrEC and LNCaP cells by 1.9 fold but not in PC3 cells (androgen independent) (Figure 1B). This suggests that SP-D expression is regulated by androgens (DHT) in androgen dependent cancer. Increased cell viability was observed in DHT treated PrEC (111.43 ± 4.69%) and LNCaP (119.23 ± 2.68%) cells as reported [Figure 1B (ii)]. *In silico* analysis of promoter region of human SP-D gene elucidated 10 putative Androgen Responsive Elements (AREs) (Table 2), suggesting that androgens may regulate SP-D expression. The functionality of identified ARE has not been evaluated in this study.

**Table 2:** Putative Androgen Responsive Elements in the promoter region of human SP-D gene^a^.

**Sr**.		**Start**	**End**	**Anchor**	**Core Sim**.	**Matrix Sim**.	**Strand**	**Sequence**
1	ARE	904	922	913	0.898	0.938	+	ataagacctcctGTGCtcc
2	ARE	2,339	2,357	2,348	1	0.895	–	gaaatgcttaaaGTTCtaa
3	ARE	3,787	3,805	3,796	1	0.912	–	ccaaagctttgtGTTCcct
4	ARE	5,430	5,448	5,439	0.878	0.901	+	ttttttctttcaGTACttt
5	ARE	5,851	5,869	5,860	1	0.912	+	ttttttctttttGTTCctc
6	ARE	7,481	7,499	7,490	1	0.941	+	gccataccttatGTTCtgc
7	ARE	7,589	7,607	7,598	1	0.8	+	caggtgctatcTGTTgttg
8	ARE	8,303	8,321	8,312	0.875	0.945	–	gctgcacccactGTCCtgc
9	ARE	8,366	8,384	8,375	0.869	0.896	–	ccctgaccccttGTGCtct
10	ARE	9,883	9,901	9,892	0.959	0.907	–	ttctctctggctGTCCtta

a *MatInspector Genomatix v3.4 Software was used for in silico analysis of human SP-D gene promoter region −10,000 bp upstream to +1,000 bp downstream from the TSS site*.

### rfhSP-D Binds Differentially to Prostate Cancer Cells

**Figure 2:**
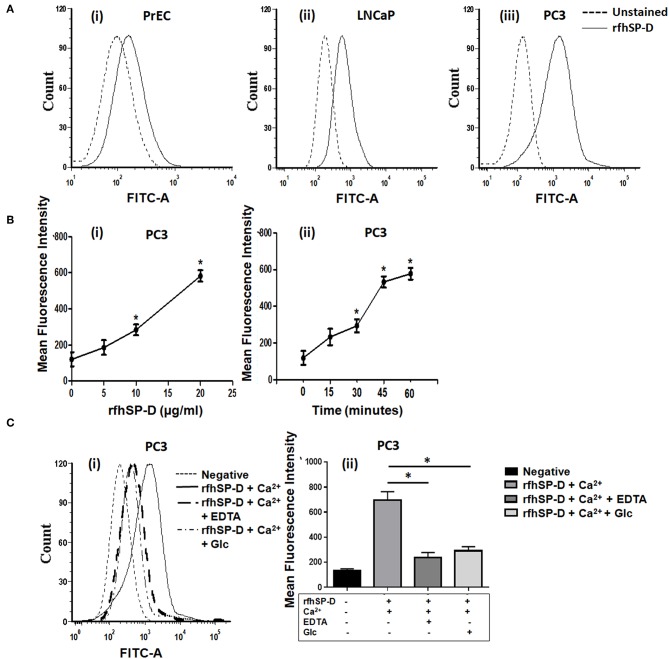
rfhSP-D differentially binds to normal prostate epithelial cells and cancer cells. **(A)** Binding of FITC labeled rfhSP-D to normal prostate epithelial cells (PrEC) and cancer cell lines (LNCaP and PC3) (representative histograms). Black dotted line represents unstained cells, black line shows rfhSP-D (20 μg/ml) binding to (i) PrEC, (ii) LNCaP, (iii) PC3 in comparison to their respective unstained. Each experiment was repeated three times. **(B)** Mean Fluorescence Intensity (MFI) of FITC labeled rfhSP-D binding to PC3 cells (i) at different doses of rfhSP-D and, (ii) time intervals. Data represents mean ± S.D of three independent experiments. *p < 0.05, vs. unstained cells. **(C)** Involvement of CRD region of rfhSP-D in its interaction with PC3 cells. (i) PC3 cells were incubated with FITC labeled rfhSP-D alone and in the presence of 5 mM Calcium (Ca^2+^) or 5 mM Ca^2+^ and 5 mM EDTA (EDTA) or 5 mM Ca^2+^ and 5 mM Glucose (Glc). A representative FACS histogram of PC3 cells with FITC-labeled rfhSP-D (20 μg/ml) showing decreased binding in presence of EDTA and glucose. (ii) Data are Mean ± S.D. of the mean fluorescence intensity of rfhSP-D binding to the PC3 cells relative to control; *n* = 3 independent experiments. *p < 0.05.

FITC-labeled rfhSP-D showed a dose and time dependent binding to PrEC and prostate cancer cells (LNCaP, DU145, and PC3) (Figure 2). A significantly higher binding was observed with the androgen independent PC3 cells (MFI-743.86 ± 67.41) than the androgen dependent LNCaP cells (MFI-354.75 ± 54.11) (*p* < 0.05) (Figure 2A). PrEC (MFI-96.48 ± 21.07) showed comparatively less binding with the FITC-labeled rfhSP-D than any of the cancer cells (PC3, LNCaP, *p* < 0.05). Binding of rfhSP-D to all cell types was calcium- and carbohydrate-dependent that was inhibitable by EDTA and glucose (Figures 2B,C show representative binding to PC3 cells). DU145 cells showed results similar to that of PC3 cells (data not shown).

### Anti-prostate Cancer Activity of rfhSP-D in PrCEC and Tumor Explants

**Figure 3:**
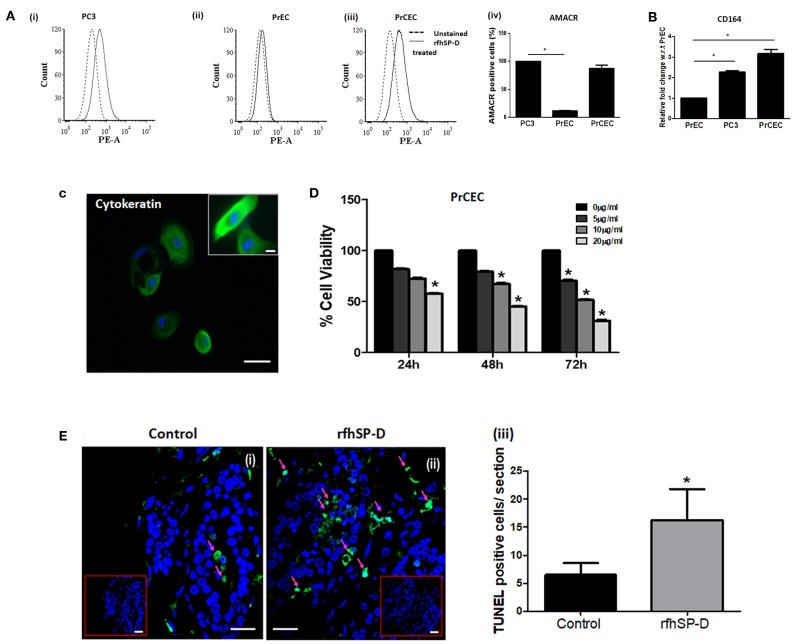
rfhSP-D reduced viability of primary Cancer Epithelial Cells (PrCEC) isolated from the metastatic prostate cancer tissue biopsies and enhanced apoptosis in tumor explants. **(A)** FACS analysis of PE tagged anti-AMACR (prostate cancer marker) expression in PrEC (*n* = 3) (i), PC3 (*n* = 3) (ii), and isolated PrCEC (*n* = 9) (iii) cell population (representative histograms). Dotted black line histogram represents isotype control. The black line histogram indicates AMACR expression. Data (iv) represents mean of positively stained AMACR cell population (%), error bar represents the S.D. *p < 0.05 vs. PC3. **(B)** Transcript levels of CD164 (marker for metastatic epithelial cell cancer) in PrEC (*n* = 3), PC3 (*n* = 3), and PrCEC (*n* = 9). Data represents the mean of three independent experiments, error bar represents the S.D. *p < 0.05 vs. PrEC. **(C)** Positive immunofluorescence staining for FITC tagged anti-Cytokeratin, a marker for epithelial cells. Inset: Higher magnification, Scale bar = 20 μm. **(D)** PrCEC were incubated with different concentrations of rfhSP-D (5, 10, or 20 μg/ml) for the indicated time intervals (24, 48, and 72 h) (*n* = 5). Each bar represents % viability ± S.D, *p < 0.05, relative to untreated controls. **(E)** TUNEL assay of tissue biopsies from metastatic prostate cancer patients exogenously treated with rfhSP-D (40 μg/ml) for 48 h. Untreated tissue biopsy (i) showed significantly less number of TUNEL positive cells when compared with the treated biopsy, (ii) from the same individual. Scale bar, 20 μm. (iii) TUNEL+ve cells were quantitated and are shown as the number of TUNEL+ve cells per section (six sections per tissue biopsy). *p < 0.05, relative to untreated controls.

We first carried out a pilot investigation on the effect of rfhSP-D on the primary cells derived from tissue biopsies of metastatic PCa patients (*n* = 9). Isolated PrCEC (Passage 2) showed positive staining for Cytokeratin (Figure 3C). Expression of AMACR (which is upregulated in prostate cancer with high-grade prostatic intraepithelial neoplasia) in PrCEC was not significantly different than the PC3 cells whereas PrEC cells showed significantly decreased expression (Figure 3A). PrCEC and PC3 cells showed significantly upregulated expression of CD164 in comparison with PrEC (Figure 3B). PrCEC cells isolated from the metastatic PCa patients showed significantly reduced viability in a dose- and time-dependent manner following treatment with rfhSP-D (Figure 3D). Importantly, TUNEL assay confirmed increased apoptosis in rfhSP-D treated tissue biopsies of the same patients (Figure 3E). Since these metastatic PCa patients were not evaluated for androgen-dependency, we could not assess if rfhSP-D induced cancer cell death would vary with androgen resistance. Therefore, we pursued further studies to delineate the molecular mechanisms for rfhSP-D mediated apoptosis in androgen dependent (LNCaP) and independent (PC3) cancer cell lines.

### rfhSP-D Selectively Reduced the Viability of Prostate Cancer Cells

**Figure 4:**
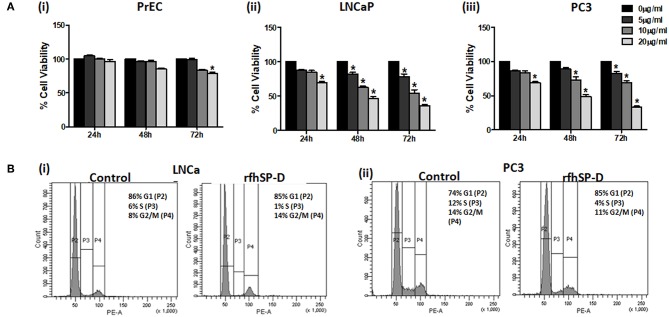
rfhSP-D treated LNCaP and PC3 cells showed increased cell cycle arrest. **(A)** (i) Normal prostate epithelial cells (PrEC); prostate cancer cell lines, LNCaP (ii) and PC3 (iii) were incubated with different concentrations of rfhSP-D (5, 10, or 20 μg/ml) for the indicated time intervals (24, 48, and 72 h). Each bar represents % viability ± S.D. of three independent experiments. *p < 0.05, relative to untreated controls. **(B)** (i) LNCaP (p53^+/+^) cells and (ii) PC3 (p53^−/−^) cells were incubated in the presence of rfhSP-D (20 μg/ml) or in culture medium alone (control) for 48 h and were subjected to flowcytometric cell cycle analysis. The figure is a representative histogram of one of the three independent experiments. The total population of cells has been divided in the G1, S, and G2 phases and analyzed using BD FACS DIVA software. Treated LNCaP (p53+/+) cells showed growth arrest in G2/M phase (14.8 ± 1.7%) in comparison with the untreated cells (8.36 ± 1.58%) (*p* < 0.05). Treated PC3 (p53^−/−^) cells showed growth arrest in G0/G1 phase (79.3 ± 3.02%) in comparison with the untreated cells (69.6 ± 4.50%) (*p* < 0.05).

Reduction in the viability of PCa cells following rfhSP-D treatment was dose- and time-dependent, irrespective of their androgen sensitivity whereas the viability of rfhSP-D treated PrEC was unaltered till 48 h (Figure 4A). The half-maximal inhibitory concentration (IC_50_) of rfhSP-D against the PCa cells is tabulated as Table 3.

**Table 3:** IC_50_ values of rfhSP-D induced cell death in various prostate cancer cells at 48 h.

**Prostate cancer cells**	**IC_**50**_ for rfhSP-D (μg/ml)**
PrEC	94.54 ± 3.97
LNCaP	23.14 ± 3.70
PC3	31.98 ± 3.42
DU145	24.80 ± 2.94

### rfhSP-D Caused Blockade in the Cell Cycle of Prostate Cancer Cells

We further analyzed the effect of rfhSP-D on the percentage of cells in various phases of the cell cycle (Figure 4B). rfhSP-D treatment significantly reduced the S-phase peak with an accumulation of cancer cells in either G0/G1 or G2/M cell cycle phases. At 48 h, we observed growth arrest of LNCaP (p53^+/+^) cells in G2/M (14.8 ± 1.7%) as compared to the untreated control (8.36 ± 1.58%) (*p* < 0.05). There was a significant increase (79.3 ± 3.02%) in G0/G1 population in the rfhSP-D treated PC3 (p53^−/−^) cells, in comparison with the untreated cells (69.6 ± 4.50%) (*p* < 0.05). Cell cycle inhibition following treatment with rfhSP-D was consistent with the cell viability results. No cell cycle arrest was observed in the PrEC (data not shown), which confirmed that the rfhSP-D mediated cell cycle arrest was specific to cancer cells.

### rfhSP-D Induced Apoptosis in LNCaP and PC3 Cells

**Figure 5:**
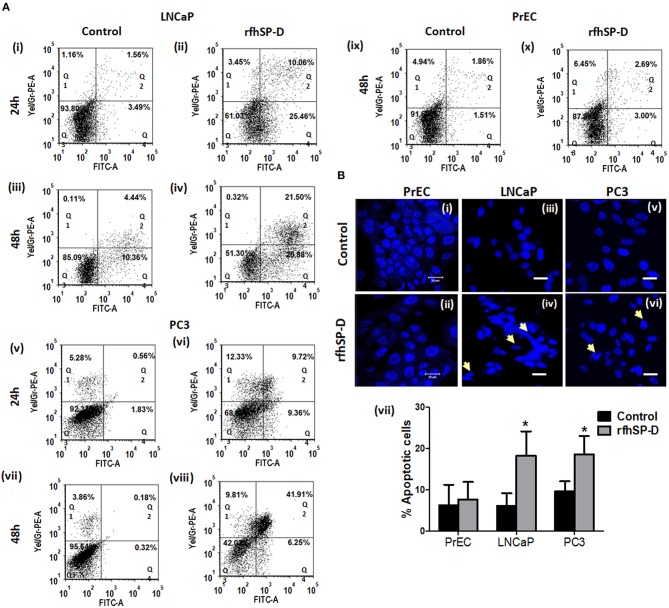
Significant increase in apoptosis in rfhSP-D treated prostate cancer cells. **(A)** Dot blots showing Annexin V-FITC and PI staining of PrEC, LNCaP, and PC3 cells incubated with rfhSP-D (20 μg/ml) for 24 and 48 h. Q1 represents necrotic cells, Q2 show Annexin V FITC and PI double positive cells in late apoptosis, Q3 is unstained cells and Q4 represents Annexin V FITC positive cells in early apoptosis. (i) LNCaP 24 h control, (ii) LNCaP 24 h rfhSP-D treated, (iii) LNCaP 48 h control, (iv) LNCaP 48 h rfhSP-D treated, (v) PC3 24 h control, (vi) PC3 24 h rfhSP-D treated, (vii) PC3 48 h control, (viii) PC3 48 h rfhSP-D treated, (ix) PrEC 48 h control, (x) PrEC 48 h rfhSP-D treated. The figure shows representative histograms from one of the three independent experiments. The total cell population has been divided into four quadrants and analyzed using FCS Express 6 software. **(B)** Fluorescence microscopy images showed that rfhSP-D promotes karyorrhexis of LNCaP and PC3 cells at 48 h after staining with highly diluted Hoechst DNA stain (1 μg/ml). (i), (iii), and (v) represent normal nuclear morphology of PrEC, LNCaP, and PC3 cells; (ii), (iv), and (vi) represent effect of rfhSP-D on nuclear morphology of PrEC, LNCaP, and PC3 cells. “White arrows” indicate disintegrated nucleus. Scale bar, 20 μm. (vii) Percent apoptotic cells was recorded in all the cell types with and without rfhSP-D treatment, each bar represents the mean ± S.D. of three independent experiments. *p < 0.05 relative to untreated controls.

Annexin-V, a classical marker of the apoptosis, was evaluated by flow cytometry. A significant increase in the annexin-V positive cells was observed post 48 h in rfhSP-D treated LNCaP (19.13 ± 2.69%[Q2] and 25.82 ± 1.14% [Q4]) (*p* < 0.05) and PC3 (40.32 ± 2.24% [Q2] and 7.405 ± 1.63% [Q4]) (*p* < 0.05) cells as compared to treated PrEC (2.63 ± 1.41% [Q2] and 3.9 ± 1.95% [Q4]) (Figure 5A). rfhSP-D promoted karyorrhexis (fragmentation of nucleus) of LNCaP and PC3 cells at 48 h, as seen in Hoechst stained cells (Figure 5B).

### rfhSP-D Triggered Intrinsic Mitochondrial Apoptosis in the Prostate Cancer Cells

**Figure 6:**
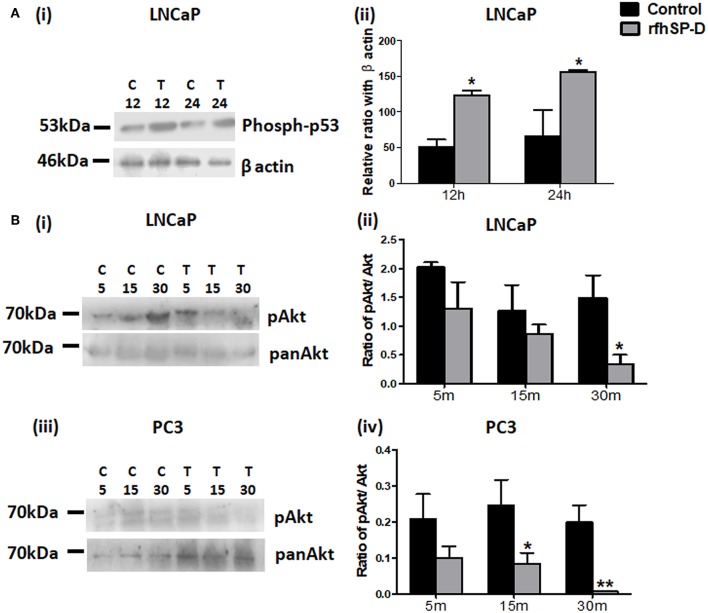
Levels of p53 and pAkt were altered in rfhSP-D treated prostate cancer cells. **(A)** LNCaP cells (i) incubated with rfhSP-D (20 μg/ml) for 12 and 24 h were analyzed for the levels of anti-phosphorylated-p53(Ser 15) (Cell Signaling Technology) by immunoblotting. Densitometric analysis for the same is shown in panel, (ii) *n* = 3 independent experiments. *p < 0.05 vs. control. **(B)** LNCaP (i) and PC3 (iii) cells treated with rfhSP-D (20 μg/ml) for 5, 15, and 30 min were analyzed for anti-pAkt (PathScan Multiplex Western Cocktail I) and anti-pan Akt (ABclonal) expression by western blot. Their respective densitometric analysis is shown as pAkt/Akt ratio in (ii) and (iv). *n* = 3 independent experiments. *p < 0.05, **p < 0.01 vs. control. Full length western blots provided as Supplementary Information.

**Figure 7:**
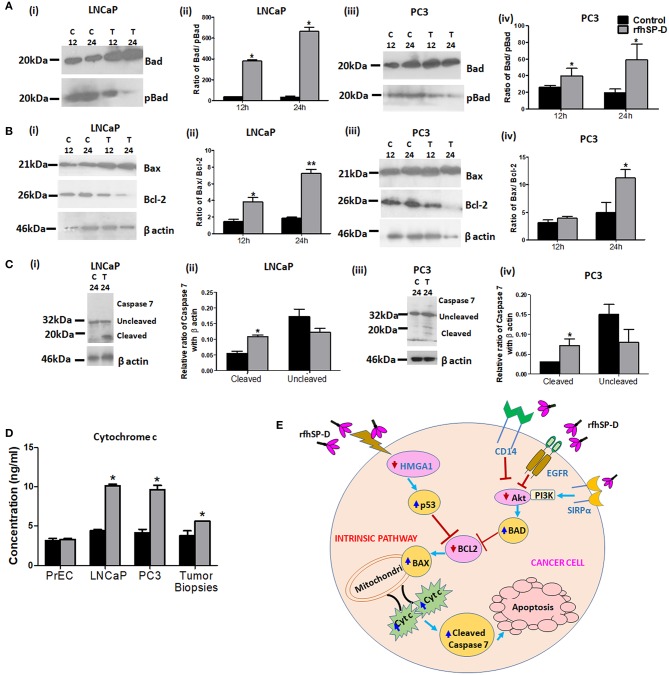
rfhSP-D triggers intrinsic apoptotic pathway. **(A)** LNCaP (i) and PC3 (iii) cells treated with rfhSP-D (20 μg/ml) for 12 and 24 h were analyzed for anti-pBad (Ser 155) (ABclonal) and anti-Bad (Apoptosis I sampler Kit, Cell Signaling Technology) expression by immunoblotting. Panels (ii) and (iv) show densitometric analysis for the Bad/pBad ratio in LNCaP and PC3 cells respectively. *n* = 3 independent experiments. *p < 0.05 vs. control. **(B)** LNCaP (i) and PC3 (iii) cells treated with rfhSP-D (20 μg/ml) for 12 and 24 h were analyzed for anti-Bax (Apoptosis I sampler Kit, Cell Signaling Technology) and anti-Bcl-2 (Apoptosis I sampler Kit, Cell Signaling Technology) expression by immunoblotting. Panels (ii) and (iv) show densitometric analysis for the Bax/Bcl2 ratio in LNCaP and PC3 cells, respectively. *n* = 3 independent experiments. *p < 0.05 vs. control, **p < 0.01 vs. control. **(C)** Immunoblotting for uncleaved anti-Caspase 7(Cell Signaling Technology) and its cleaved products of molecular mass 32 kDa (upper panel; black arrow) and 20 kDa (lower panel; black arrow) respectively, in rfhSP-D (20 μg/ml) treated LNCaP (i) and PC3 (iii) cells for 24 h. Panels (ii) and (iv) show densitometric analysis for the cleaved caspase 7 and uncleaved caspase 7 in rfhSP-D treated LNCaP and PC3 cells, respectively. *n* = 3 independent experiments. *p < 0.05 vs. control. Full length western blots provided as Supplementary Information. **(D)** Measurement of Cytochrome c (Human Cyt-C, catalog no. E1516Hu, BT Assay) in cell culture supernatants of normal prostate epithelial cells (PrEC), cancer cell lines (LNCaP and PC3) and tumor tissue biopsies on exposure to rfhSP-D treatment for 48 h by ELISA. Data are presented as the mean ± S.D from three independent experiments. *p < 0.05 vs. control. **(E)** Proposed mechanisms for rfhSP-D mediated apoptosis in PCa cells. Treatment with rfhSP-D upregulates p53 and downregulates pAkt, resulting in upregulation of Bad, Bax, and release of cytochrome c leading to cleavage of caspase 7 in prostate cancer cells. SP-D interaction with some key molecules like HMGA1, CD14, SIRPα, and EGFR has been reported previously and may be relevant as part of the proposed mechanisms of p53 and Akt.

**Figure 8:**
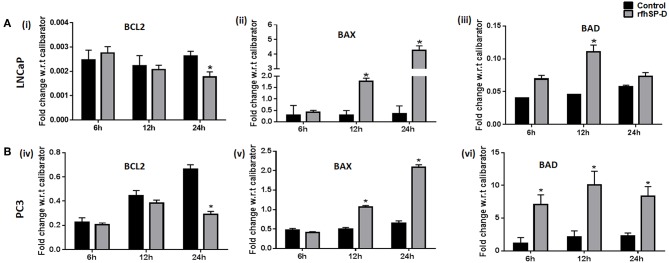
rfhSP-D treatment induced alterations in the transcripts of anti and pro-apoptotic genes. **(A,B)** Real time RT-PCR analysis showed an upregulation of transcripts of BAX (ii, v) BAD (iii, vi) and downregulation of BCL2 (i, iv) in LNCaP and PC3 cells on treatment with rfhSP-D (20 μg/ml) for 6, 12, and 24 h. Each bar represents the mean ± S.D of three independent experiments. * indicates statistical significance and *p < 0.05 relative to rfhSP-D untreated controls.

p53 and PI3K/Akt are involved in several cellular physiological processes including apoptosis and cell proliferation. Our previous study had shown involvement of p53 in the rfhSP-D mediated apoptosis of AML14.3D10 cells (12). Here, rfhSP-D treated LNCaP cells with a wild type p53, showed significant upregulation of p53 (Figure 6A). To understand the other likely mechanisms of apoptosis, p53 null PC3 cells were examined (Figure 6B) for differential expression of several kinases (MAPK, JAK, Stat-1, ERK, and Akt). rfhSP-D significantly decreased the phospho-active forms of Akt in PC3 cells at 15 min (Figure 6B iii and iv). Interestingly, treated LNCaP cells also showed significantly decreased pAkt at 30 min (Figure 6B i and ii). Phosphorylated Akt leads to increased phosphorylation of Bad, an inactive form of Bad. The rfhSP-D induced decrease in pAkt sequentially led to a significant decrease in phosphorylated Bad in both the cell lines (Figure 7A). rfhSP-D treatment further led to a significantly decreased level of Bcl2 and an increased level of Bax at 24 h (Figure 7B). Both LNCaP and PC3 cells showed a significant increase in Bax to Bcl-2 ratio (*p* < 0.05) upon treatment with rfhSP-D. Similarly, transcripts for pro-apoptotic genes like BAX, BAD were upregulated and transcripts of BCL2 an anti-apoptotic gene was down regulated in a time dependent manner in LNCaP and PC3 cells (Figure 8). In LNCaP cells, transcripts for BAX (by 1.5 folds) were upregulated during 12 h of rfhSP-D incubation; in PC3 cells, transcripts for BAD (by 6 folds) were significantly upregulated at 6 h of rfhSP-D incubation.

To delineate the penultimate steps of apoptotic cascade, we assessed levels of cytochrome c and activation of caspases in rfhSP-D treated prostate cancer cells. At 48 h, rfhSP-D treatment induced release of cytochrome c attained significance in culture supernatant of cancer cells (LNCaP, PC3) and tissue biopsies of prostate cancer when compared to PrEC (Figure 7D). Cleaved products of the executioner caspase 7 were significantly increased after 24 h following rfhSP-D treatment (Figure 7C). From these observations, we infer that rfhSP-D triggers intrinsic mitochondrial pathway of apoptosis in prostate cancer cells.

## Discussion

The present study established the anti-prostate cancer activity of rfhSP-D via induction of intrinsic apoptosis in explants and primary tumor cells isolated from tissue biopsies of metastatic prostate cancer patients and prostate cancer cell lines: LNCaP (androgen responsive) and PC3 (androgen resistant). Various attributes of apoptosis like phosphatidylserine externalization (Figure 5), mitochondrial dysfunction (Figure 7), and DNA fragmentation (Figure 5) and various apoptotic markers (Figure 7) were observed in the rfhSP-D treated LNCaP and PC3 cells. Viability of the normal prostate epithelial cells (PrEC) was not altered in presence of rfhSP-D (Figure 1).

Human prostate cancer tissues frequently exhibit inactivation of the tumor suppressor gene p53 which is associated with therapeutic resistance (23). The p53 pathway plays a crucial role in the transmission of pro-apoptotic signals (24). Various therapeutic agents/candidates induce apoptosis of LNCaP (p53^+/+^) cells by increasing the levels of phosphorylated p53 (25–27). Knockdown of p53 resulted in blockade of docetaxel induced apoptotic cell death in prostate cancer cells (28). Upregulation of activated p53 levels in a time-dependent manner by rfhSP-D treatment suggested involvement of the p53 pathway in the induction of apoptosis of LNCaP cells. Previously, activation of p53 was also observed in the eosinophilic leukemic cells (AML14.3D10) undergoing apoptosis upon treatment with rfhSP-D (14, 29). rfhSP-D treated AML14.3D10 cells showed significantly reduced levels of HMGA1, a survival protein (14). PC3 cells, a p53 null and highly metastatic prostate cancer cell line, also showed significant apoptosis following treatment with rfhSP-D, which suggested involvement of a p53 independent mechanism of apoptosis. Among 25% prostate cancer cases, diallelic deletion of the Phosphatase and tensin homolog (PTEN) gene and the associated increase in Akt phosphorylation correlates with hormone refractory prostate cancer (30). Decreased levels of activated Akt may lead to decreased levels of phosphorylated Bad (Bcl-2 associated death promoter). Dephosphorylated Bad interferes with interaction of activated Bcl2 with Bax. Thus, increased release of Bax triggers apoptosis (31, 32). Decreased ratio of transcripts of *Bcl-2* to *Bax* has been associated with cell death following an apoptotic stimulus (33, 34). Both LNCaP and PC3 cells, upon treatment with rfhSP-D, showed a significant increase in Bax to Bcl-2 ratio (*p* < 0.05), suggesting that besides activation of p53 pathway, rfhSP-D also inhibited Akt-PI3K pathway leading to induction of Bax mediated apoptosis. The present study unravels PI3K/Akt, an anti-apoptotic pathway, as a novel target of rfhSP-D mediated anti-prostate cancer activity.

Mitochondria plays a central role in the initiation of intrinsic apoptosis pathway (35). rfhSP-D treatment mediated disruption of the mitochondria was elucidated by its reduced ability to oxidize methyl tetrazolium to form formazan crystals (MTT assay; Figure 4). Breakdown of mitochondria is followed by the release of cytochrome c (Figure 7), a key initial step in the irreversible apoptotic process (35, 36). Culture supernatants from the rfhSP-D treated prostate cancer cells and cancer tissue biopsies showed significantly elevated levels of cytochrome c, confirming induction of intrinsic apoptosis. Once in the cytosol, the cytochrome c interacts with its adaptor molecule, Apaf-1, resulting in the processing and activation of pro-caspase-9 (37). Caspase-9, in turn, cleaves and activates pro-caspases−3 and−7, effector caspases responsible for the cleavage of various proteins leading to the biochemical and morphological features observed in apoptosis (38). Treatment of the prostate cancer cells with rfhSP-D resulted in the cleavage and activation of the effector caspase-7, thus leading to programmed cell death. Nuclear condensation, membrane and nuclear blebbing and flipping of phosphatidylserine (PS) on the cell membranes, the typical morphological characteristics of apoptotic cells, were observed in the rfhSP-D treated prostate cancer cells, confirming apoptotic cell death. The proposed mechanisms for the rfhSP-D mediated apoptosis in prostate cancer cells as revealed in this study along with some key molecules of these pathways interacting with/ induced by SP-D as reported previously have been depicted in Figure 7E.

Recent studies highlighted the suppressive role of SP-D in extrinsic apoptosis wherein native purified human SP-D interacted with Jurkat T cells and delayed the progression of Fas (CD95)-Fas ligand and TRAIL-TRAIL receptor induced, but not TNF-TNF receptor-mediated apoptosis (39). In a subsequent study from the same group in UV irradiated Jurkat T cells, SP-D reduced the activation of caspase-8, executioner caspase-3 and exposure of phosphatidylserine (PS) on the membranes of dying cells, with a concomitant increase in the formation of nuclear and membrane blebs (16). The involvement of rfhSP-D in extrinsic apoptotic pathway in prostate cancer cells needs to be explored further.

rfhSP-D induced a cell cycle arrest in G2/M and G0/G1 phases of LNCaP and PC3 cells, respectively. We had previously shown that the rfhSP-D induced elevated p21 expression and inhibited Cdc2 phosphorylation resulting in reduced activity of Cdc2-cyclin B1 thus, leading to G2/M arrest in the AML14.3D10 cells (14). Several genes are common to the pathways involved in cell cycle regulation and apoptosis (40). The p53 protein plays a critical role both in the G1/S and G2/M checkpoint while Retinoblastoma (Rb) protein is a potent inhibitor of the G1 to S phase transition in the cell cycle (41, 42). Following rfhSP-D treatment, PC3 cells, which are null p53 but express the wild type Rb gene, showed an arrest in the G1-phase with significant reduction in the S-phase cell population. This alluded to the involvement of a p53 independent mechanism in the rfhSP-D induced apoptosis in PC3 cells.

Increased interaction of rfhSP-D with the prostate cancer cells as compared to PrEC, could be due to differential expression of the receptors. Similar to our previous study with eosinophilic leukemic cells, rfhSP-D showed interaction with the prostate cancer cells via carbohydrate recognition domain (CRD) (14). CRD domain of SP-D is known to interact with CD14, TLR-2, TLR-4, EGFR, and SIRPα that are reported to be present on prostate cancer cells and normal prostate tissues (15, 43, 44). Binding of SP-D with pattern recognition receptors CD14, TLR-2, and TLR-4 may lead to blockade of their downstream pro-inflammatory and pro-survival signaling (Figure 7E). Interaction with SIRPα, involved in the negative regulation of receptor tyrosine kinase-coupled signaling processes, may result in recruitment of PI3K, leading to a reduction in the activity of the downstream kinase Akt (45) (Figure 7E). A recent study showed that SP-D reduced EGF -EGFR binding through the interaction between CRD of SP-D and N-glycans of EGFR, thus, leading to downregulation of EGF signaling in the A549 human lung adenocarcinoma cells (15) (Figure 7E). Further studies are needed to elucidate the involvement of CRD and its interacting partners in downstream signaling of rfhSP-D in the prostate cancer cells.

Oncogenic mutations disturb the normal cellular functions, thus, allowing the tumor cells to undergo dysregulated proliferation, resist pro-apoptotic insults, invade normal tissues, and most importantly, escape apoptosis. Therefore, induction of apoptosis in the malignant tumors has evolved as a successful adjunct anti-cancer strategy. We report that rfhSP-D selectively triggered intrinsic apoptosis in androgen-dependent as well as androgen-independent prostate cancer cells, without affecting normal epithelial cells (PrEC). rfhSP-D also induced apoptotic cell death in the tissue biopsies from metastatic prostate cancer patients. Thus, SP-D is likely to act as an integral component of the human innate immune surveillance against cancer cells. A great advantage associated with the anti-cancer activity of rfhSP-D is induction of apoptosis by simultaneous targeting of multiple cellular signaling pathways including transcription factors, tumor cell survival factors, protein kinases resulting in the efficient and selective killing of prostate cancer cells. Murine models of prostate cancer including the patient-derived xenograft (PDX) mouse models that mimic human disease and 3D cell cultures derived from PDX are going to be valuable tools for the evaluation of therapeutic strategies using rfhSP-D (46).

## Data Availability

The raw data supporting the conclusions of this manuscript will be made available by the authors, without undue reservation, to any qualified researcher.

## Ethics Statement

The study was approved by the Institutional Ethics Committee for Clinical Studies, ICMR-National Institute for Research in Reproductive Health (Project No.: 260/2014) and Tata Memorial Hospital (Study no: 1467). Tissue biopsy samples were collected from 9 metastatic prostate cancer patients (treatment naïve) using Trans-rectal Ultrasound guided multiple core needle, with written informed consent. Chemotherapy was started in the case of patients following confirmation of metastasized prostate cancer. Information regarding the androgen dependency of the prostate cancer in these patients was not available. Average age of the study participants was 67.4 ± 3.97 years with Mean PSA (Prostate-specific antigen) level of 190.04 ± 85.12 ng/ml and Median Gleason score of 8 ± 0.83.

## Author Contributions

GT conceived and co-ordinated the study, designed, performed and analyzed the experiments, and wrote the paper. GP, VM, and GB designed and co-ordinated the study. GP recruited and screened the study participants. NS screened the study participants and collected tissue biopsies. SM interpreted the laboratory investigations of study participants. SA, HK, VM, and UK expressed, purified and characterized rfhSP-D for the study. UK provided purified and characterized rfhSP-D for the study and critical suggestions for the manuscript. TM conceived and co-ordinated the study, procured the intra-mural grant support, mediated the clinical collaboration, defended the protocol for IEC approval, analyzed the data, and edited the paper. All authors reviewed the results and approved the final version of the manuscript.

### Conflict of Interest Statement

The authors declare that the research was conducted in the absence of any commercial or financial relationships that could be construed as a potential conflict of interest.
