# Supplementary material for: Human SP-D Acts as an Innate Immune Surveillance Molecule Against Androgen-Responsive and Androgen-Resistant Prostate Cancer Cells
Source: Front Oncol. 2019 Jul 11;9:565. doi: 10.3389/fonc.2019.00565 (PMC6637921; doi:10.3389/fonc.2019.00565)
Supplement: Supplementary file 1 [file Data_Sheet_1.zip › supplementary datasheet 1/supplementary data legends.docx]

**Supplementary Data Figure Legends**

**Figure S1. SP-D expression in prostate cells**

Full length Western blot for SP-D expression in PrEC, DU145, PC3 and LNCaP cells as shown in Figure 2A(i) alongwith β-actin as loading control. Molecular weight ladder used is RPN800E. Total cell lysate proteins were probed with primary mouse monoclonal SP-D antibody, (1:1000, a gift from Uffe Holmskov) and β-actin mouse monoclonal antibody (1:500, ABclonal) followed by HRP- conjugated Goat anti- mouse IgG secondary antibody (1:6000, ABclonal).

**Figure S2. Phosphorylated p53 protein expression in rfhSP-D treated LNCaP cells**

Full length Western blot for Phosphorylated p53 protein expression in rfhSP-D treated LNCaP cells as shown in Figure 6A(i) alongwith β-actin as loading control. LNCaP cells were incubated with rfhSP-D (20µg/ml) for 12 h and 24 h and were analyzed for the levels of phosphorylated p53 by immunoblotting. Molecular weight ladder used is RPN800E. Total cell lysate proteins were probed with Phosphorylated-p53 (Ser 15) polyclonal antibody (1:500, Cell Signaling Technology) and β-actin mouse monoclonal antibody (1:500, ABclonal) followed by HRP- conjugated Goat anti- mouse (1:6000, Abclonal) / anti- rabbit (1:6000, Abclonal) IgG secondary antibody.

**Figure S3.** **Phosphorylated Akt and Akt protein expression in rfhSP-D treated LNCaP cells**

Full length Western blot for Phosphorylated Akt and Akt protein expression in rfhSP-D treated LNCaP cells as shown in Figure 6B(i) alongwith Ponceau stained blot used as total protein control. LNCaP cells treated with rfhSP-D (20µg/ml) for 5 min,15 min and 30 min were analysed for pAkt and Akt expression by western blot. Levels of pAkt were altered in rfhSP-D treated prostate cancer cells. Molecular weight ladder used is PG-PMT2922. Total cell lysate proteins was probed with pAKT (1:500, PathScan® Multiplex Western Cocktail I), or pan Akt (1:1000, ABclonal). The analysis was performed by taking the ratio of normalised pAkt to normalised Akt. Normalized pAkt = pAkt / total protein; Normalized Akt = Akt / total protein (Total Protein = densitometric analysis of every band seen on Ponceau staining).

**Figure S4.** **Phosphorylated Akt and Akt protein expression in rfhSP-D treated PC3 cells**

Full length Western blot for Phosphorylated Akt and Akt protein expression in rfhSP-D treated PC3 cells as shown in Figure 6B(iii) alongwith Ponceau stained blot used as total protein control. PC3 cells treated with rfhSP-D (20µg/ml) for 5 min,15 min and 30 min were analysed for pAkt and Akt expression by western blot. Levels of pAkt were altered in rfhSP-D treated prostate cancer cells. Molecular weight ladder used is PG-PMT2922. Total cell lysate proteins was probed with pAKT (1:500, PathScan® Multiplex Western Cocktail I), or pan Akt (1:1000, ABclonal). The analysis was performed by taking the ratio of normalised pAkt to normalised Akt. Normalized pAkt = pAkt / total protein; Normalized Akt = Akt / total protein (Total Protein = densitometric analysis of every band seen on Ponceau staining).

**Figure S5.** **Phosphorylated Bad and Bad protein expression in rfhSP-D treated LNCaP cells**

Full length Western blot for Phosphorylated Bad and Bad protein expression in rfhSP-D treated LNCaP cells as shown in Figure 7A(i) alongwith β-actin as loading control. LNCaP cells treated with rfhSP-D (20µg/ml) for 12 h and 24 h were analysed for pBad (Ser 155) and Bad expression by immunoblotting. Levels of pBad were upregulated in rfhSP-D treated prostate cancer cells. Molecular weight ladder used is RPN800E. Total cell lysate proteins was probed with phospho-Bad-S155 (1:500, ABclonal), or Bcl-2 associated death promoter (Bad) (1:250, Apoptosis I sampler Kit).

**Figure S6. Phosphorylated Bad and Bad protein expression in rfhSP-D treated PC3 cells**

Full length Western blot for Phosphorylated Bad and Bad protein expression in rfhSP-D treated PC3 cells as shown in Figure 7A(iii) alongwith β-actin as loading control. PC3 cells treated with rfhSP-D (20µg/ml) for 12 h and 24 h were analysed for pBad (Ser 155) and Bad expression by immunoblotting. Levels of pBad were upregulated in rfhSP-D treated prostate cancer cells. Molecular weight ladder used is RPN800E. Total cell lysate proteins was probed with phospho-Bad-S155 (1:500, ABclonal), or Bcl-2 associated death promoter (Bad) (1:250, Apoptosis I sampler Kit).

**Figure S7. Bax and Bcl2 protein expression in rfhSP-D treated LNCaP cells**

Full length Western blot for Bax and Bcl2 protein expression in rfhSP-D treated LNCaP cells as shown in Figure 7B(i) alongwith β-actin as loading control. LNCaP cells treated with rfhSP-D (20µg/ml) for 12 h and 24 h were analysed for Bax and Bcl2 expression by immunoblotting. Levels of Bax were upregulated in rfhSP-D treated prostate cancer cells. Molecular weight ladder (RPN800E) used is same as Figure S5, since the same set of experiment. Total cell lysate proteins was probed with Bcl-2-associated X protein (Bax) (1:500, Apoptosis I sampler Kit) and B-cell lymphoma 2 (Bcl2) (1:500, Apoptosis I sampler Kit).

**Figure S8.** **Bax and Bcl2 protein expression in rfhSP-D treated PC3 cells**

Full length Western blot for Bax and Bcl2 protein expression in rfhSP-D treated PC3 cells as shown in Figure 7B(iii). LNCaP cells treated with rfhSP-D (20µg/ml) for 12 h and 24 h were analysed for Bax and Bcl2 expression by immunoblotting. Levels of Bax were upregulated in rfhSP-D treated prostate cancer cells. Molecular weight ladder (RPN800E) used is same as Figure S6, since the same set of experiment. Total cell lysate proteins was probed with Bcl-2-associated X protein (Bax) (1:500, Apoptosis I sampler Kit) and B-cell lymphoma 2 (Bcl2) (1:500, Apoptosis I sampler Kit).

**Figure S9.** **Caspase 7 protein expression in rfhSP-D treated LNCaP cells**

Full length Western blot for Caspase 7 protein expression in rfhSP-D treated LNCaP cells as shown in Figure 7C(i) along with β-actin as loading control. Immunoblotting for uncleaved Caspase 7 and its cleaved products of molecular mass 32 kDa (upper panel; black arrow) and 20 kDa (lower panel; black arrow) respectively, in rfhSP-D (20µg/ml) treated LNCaP cells for 24h. Total cell lysate proteins was probed with caspase 7 (1:250, Cell Signaling Technology) and β-actin mouse monoclonal antibody (1:500, ABclonal) followed by HRP- conjugated Goat anti- mouse IgG secondary antibody (1:6000, ABclonal).

**Figure S10.** **Caspase 7 protein expression in rfhSP-D treated PC3 cells**

Full length Western blot for Caspase 7 protein expression in rfhSP-D treated PC3 cells as shown in Figure 7C(iii) along with β-actin as loading control. Immunoblotting for uncleaved Caspase 7 and its cleaved products of molecular mass 32 kDa (upper panel; black arrow) and 20 kDa (lower panel; black arrow) respectively, in rfhSP-D (20µg/ml) treated PC3 cells for 24h. Total cell lysate proteins was probed with caspase 7 (1:250, Cell Signaling Technology) and β-actin mouse monoclonal antibody (1:500, ABclonal) followed by HRP-conjugated Goat anti- mouse IgG secondary antibody (1:6000, ABclonal). Molecular weight ladder (RPN800E) used is same as Figure S6, since the same set of experiment.
