# Supplementary figures and images for: Human SP-D Acts as an Innate Immune Surveillance Molecule Against Androgen-Responsive and Androgen-Resistant Prostate Cancer Cells
Source: Front Oncol. 2019 Jul 11;9:565. doi: 10.3389/fonc.2019.00565 (PMC6637921; doi:10.3389/fonc.2019.00565)

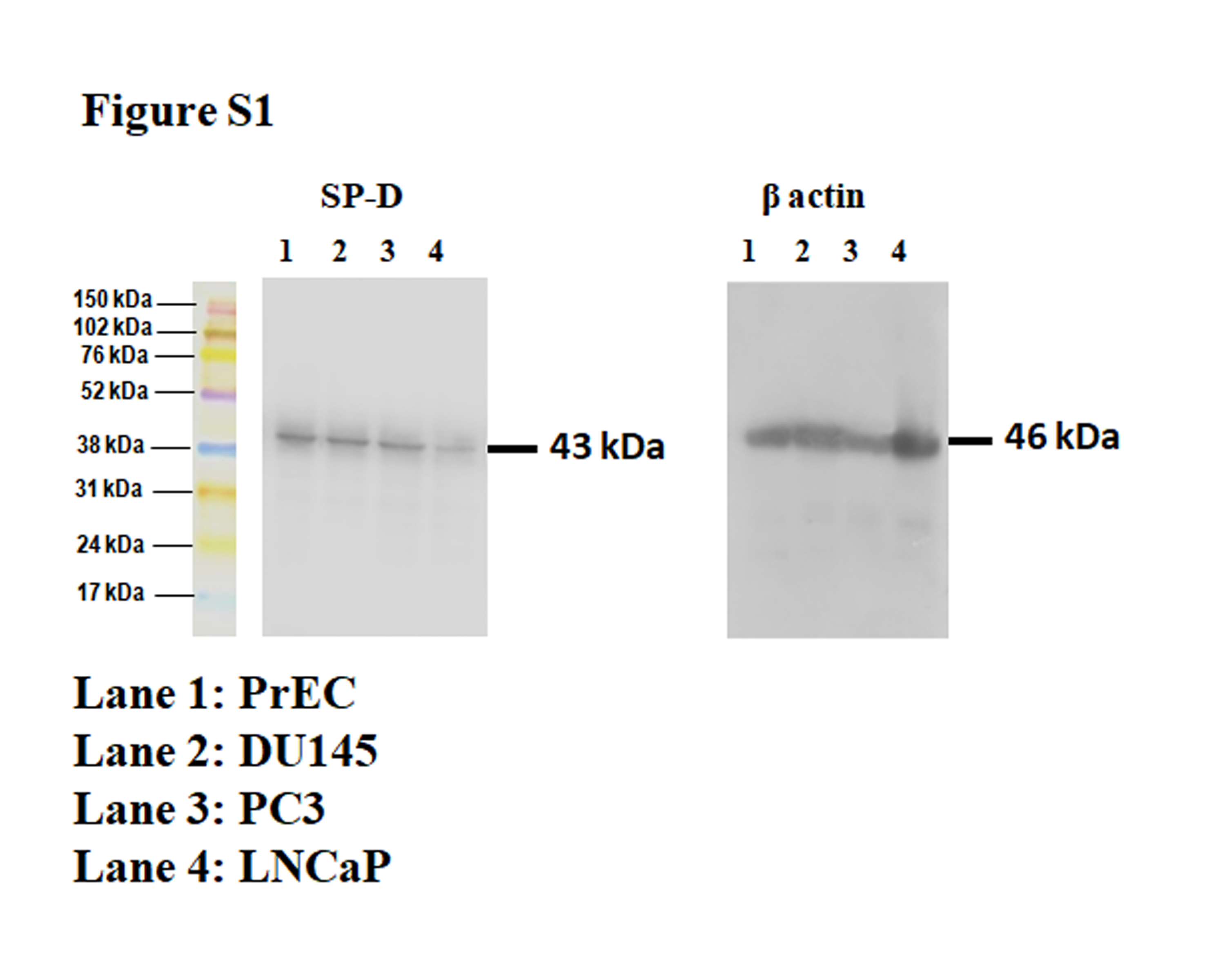

Supplement: Supplementary file 1 [file Data_Sheet_1.zip › supplementary datasheet 1/S1.tif]

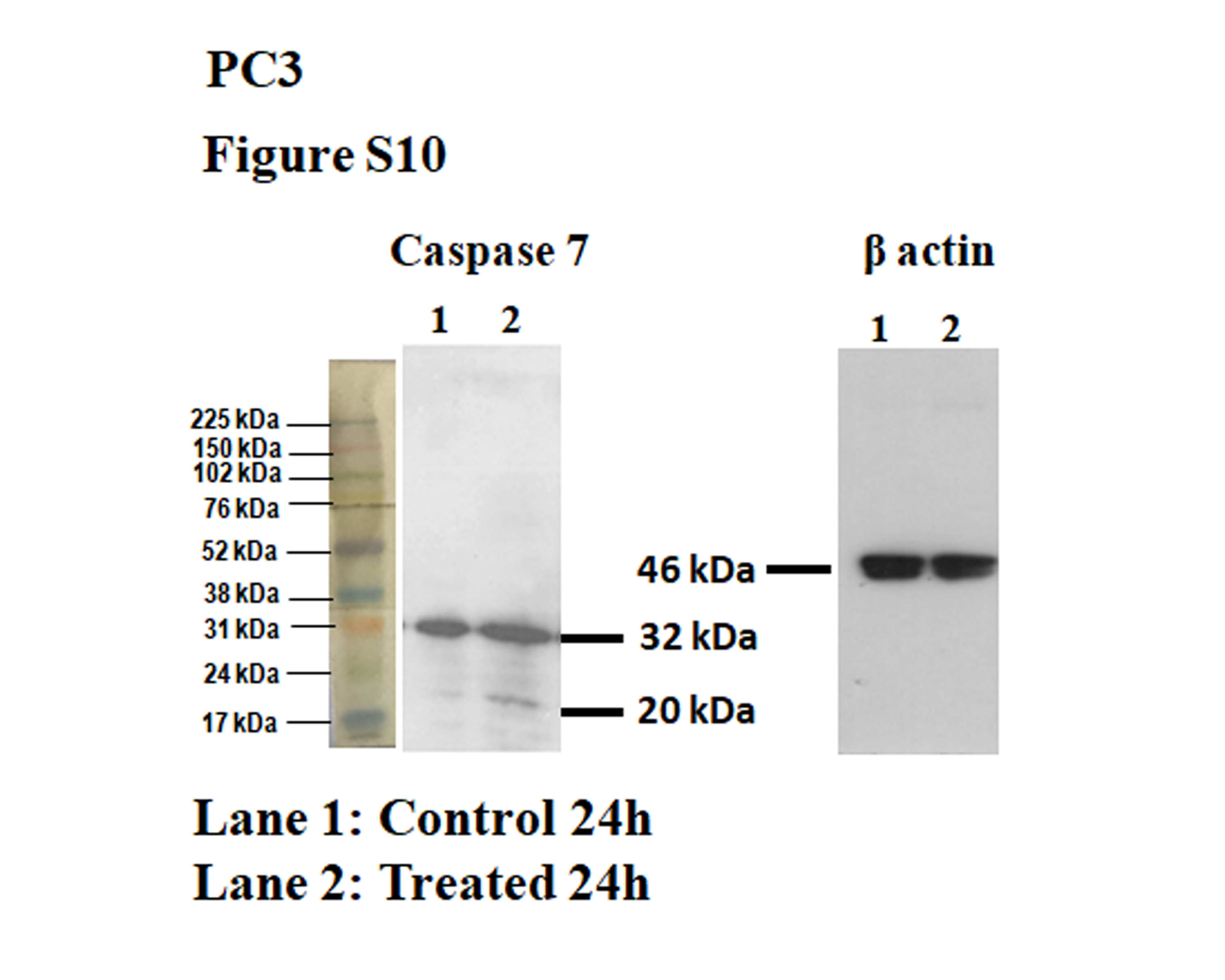

Supplement: Supplementary file 1 [file Data_Sheet_1.zip › supplementary datasheet 1/S10.tif]

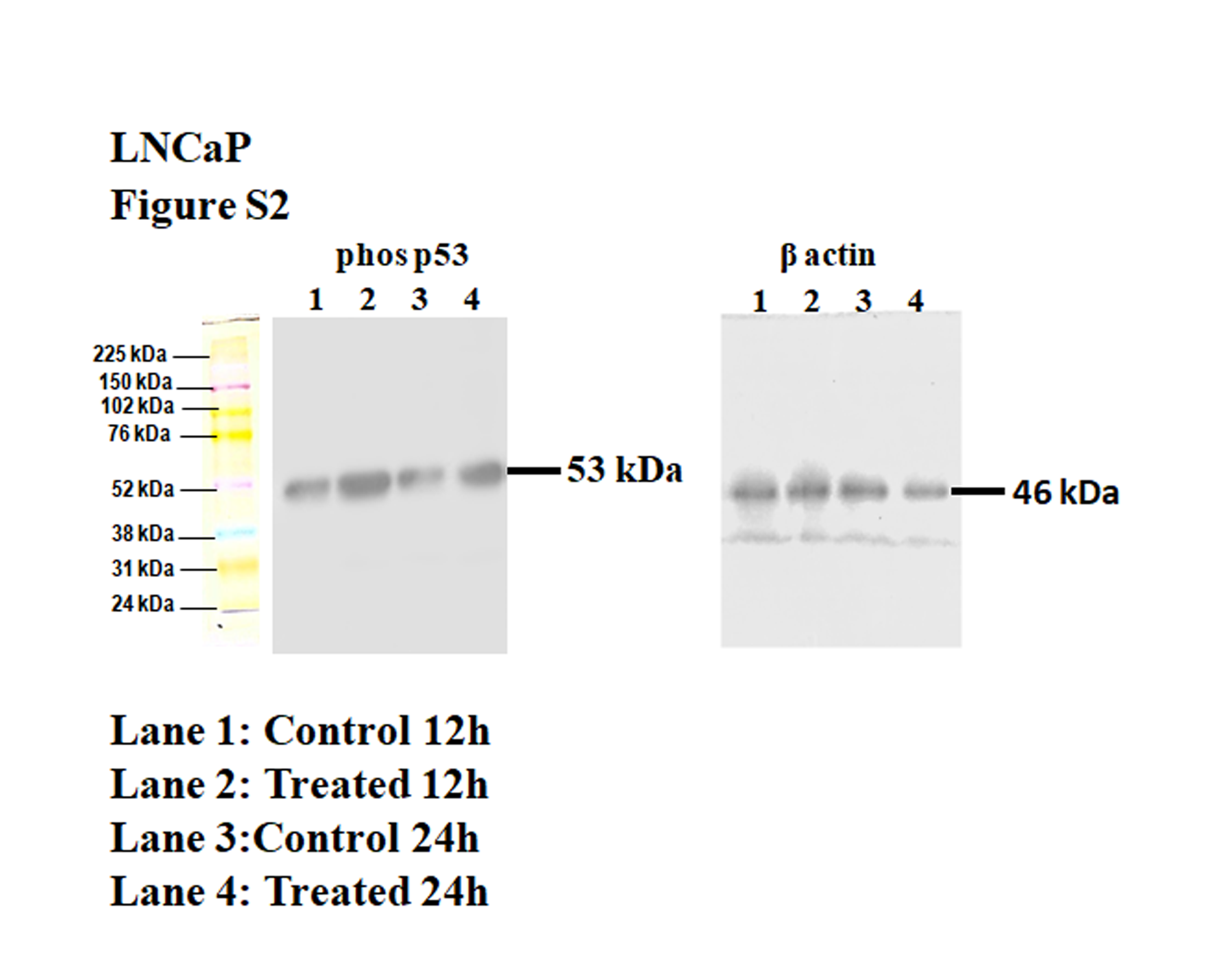

Supplement: Supplementary file 1 [file Data_Sheet_1.zip › supplementary datasheet 1/S2.tif]

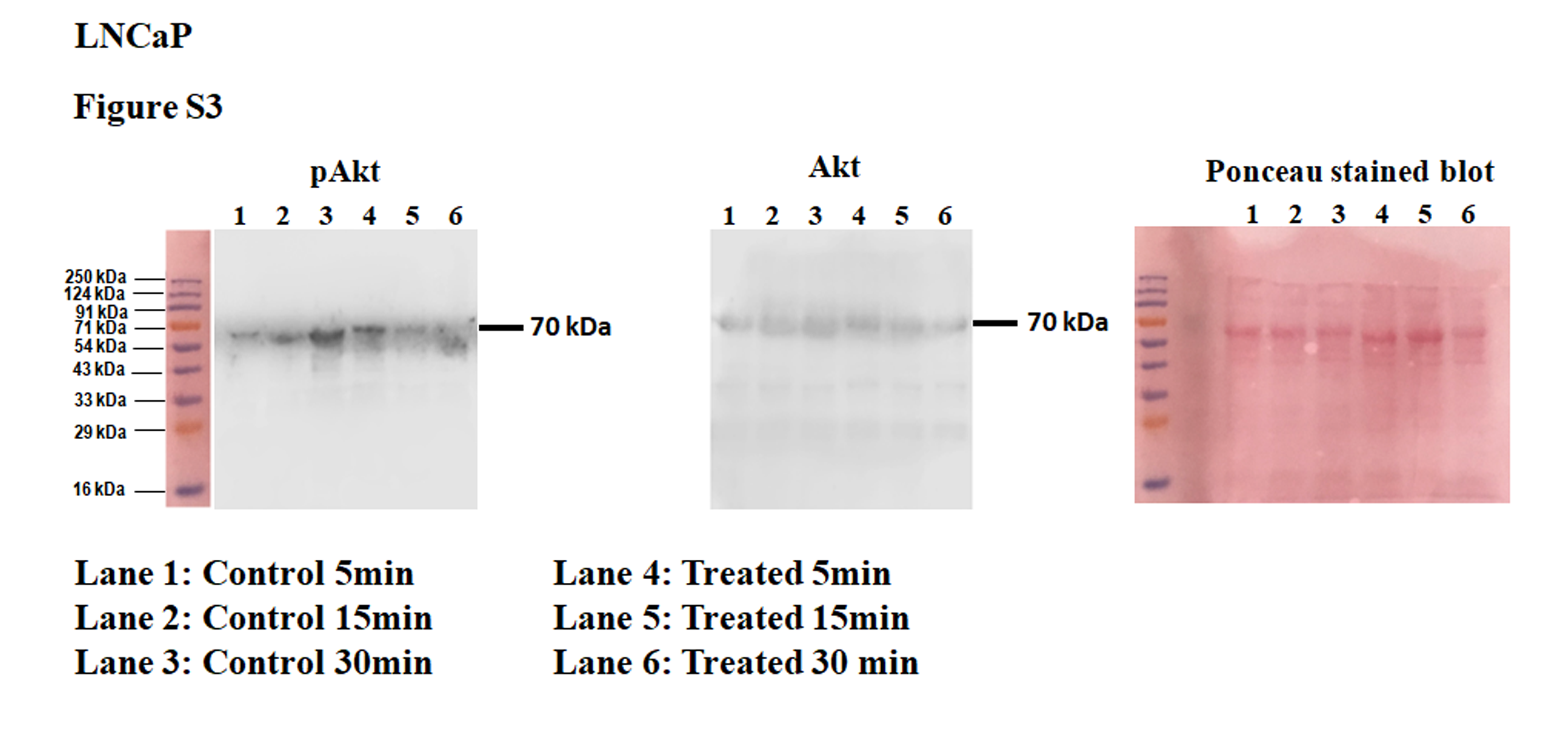

Supplement: Supplementary file 1 [file Data_Sheet_1.zip › supplementary datasheet 1/S3.tif]

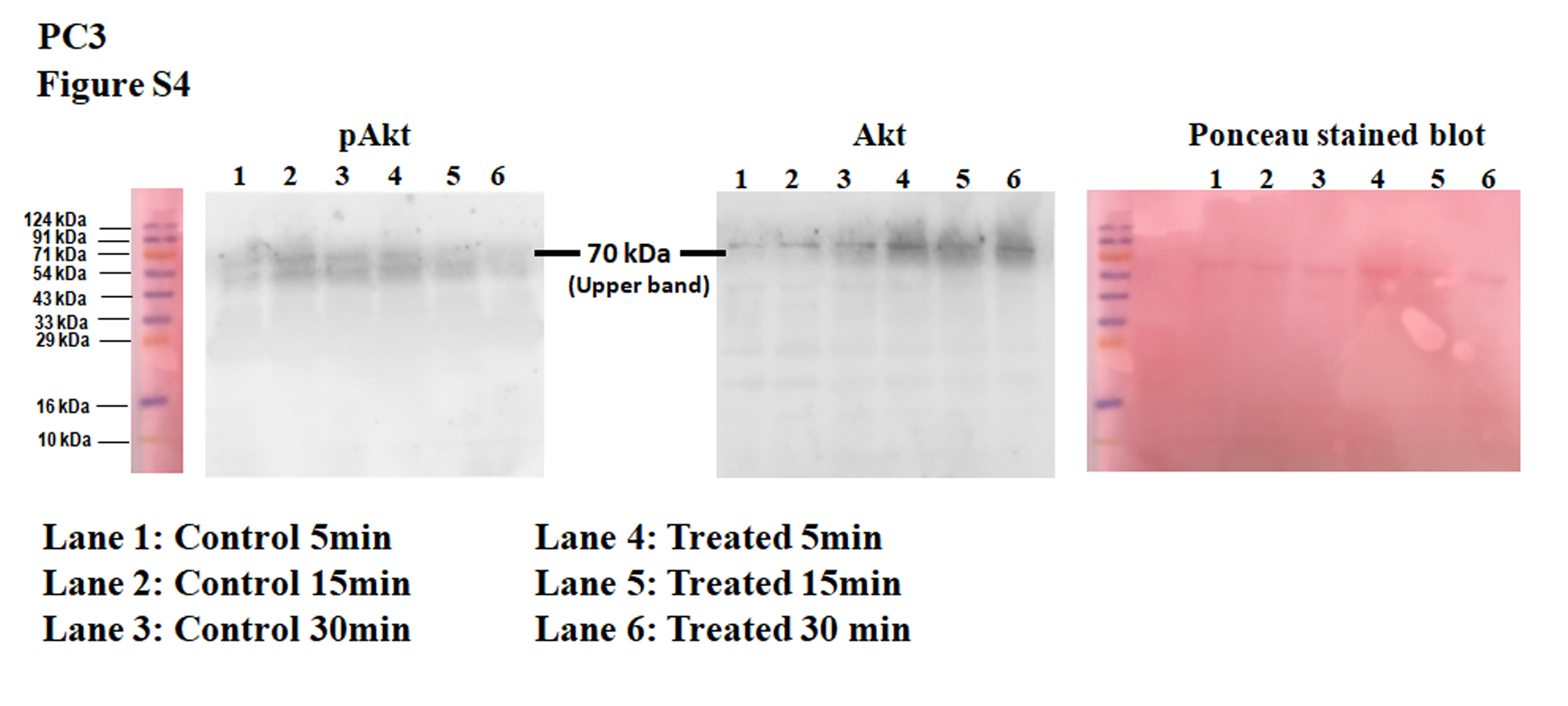

Supplement: Supplementary file 1 [file Data_Sheet_1.zip › supplementary datasheet 1/S4.tif]

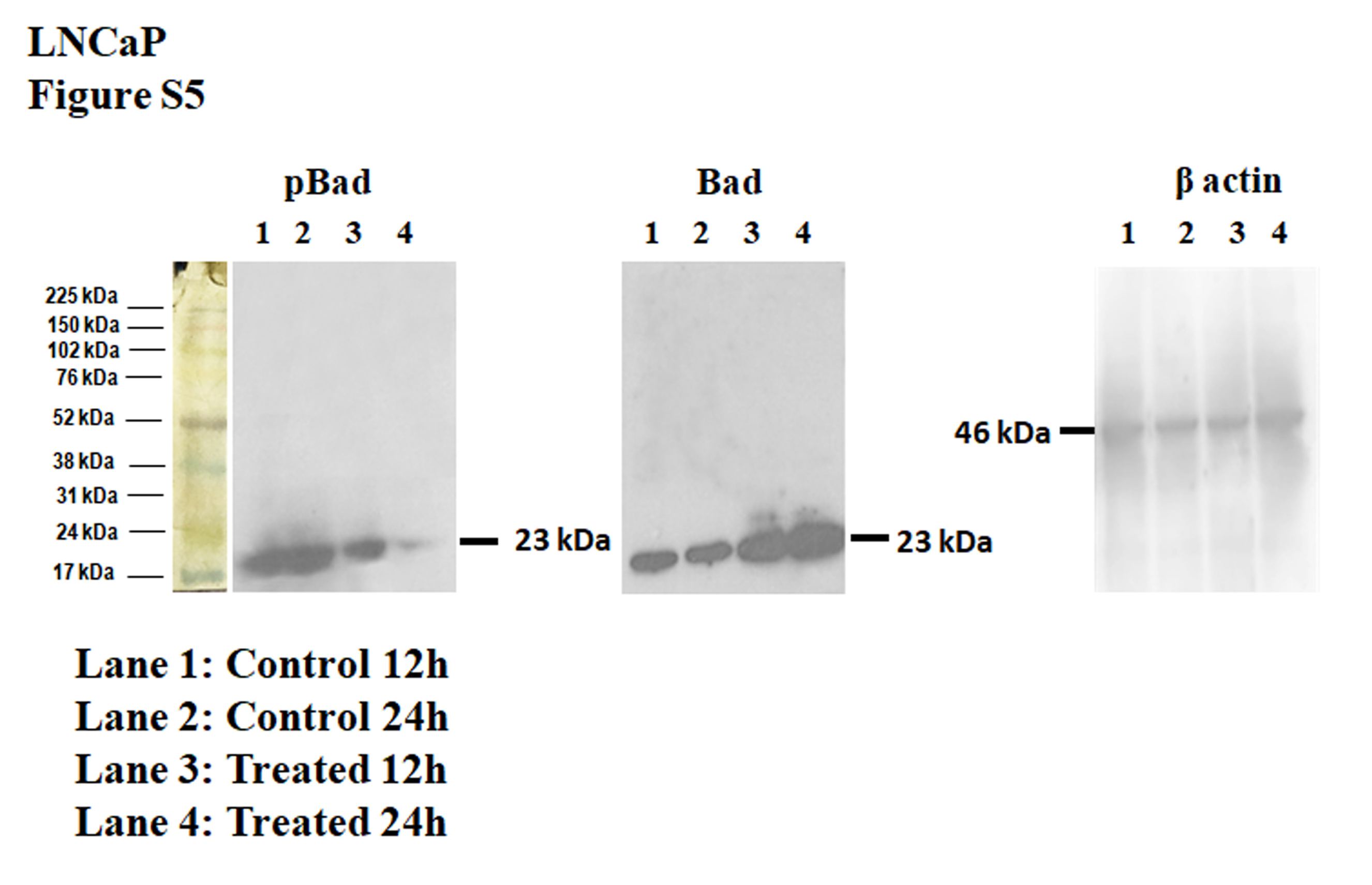

Supplement: Supplementary file 1 [file Data_Sheet_1.zip › supplementary datasheet 1/S5.tif]

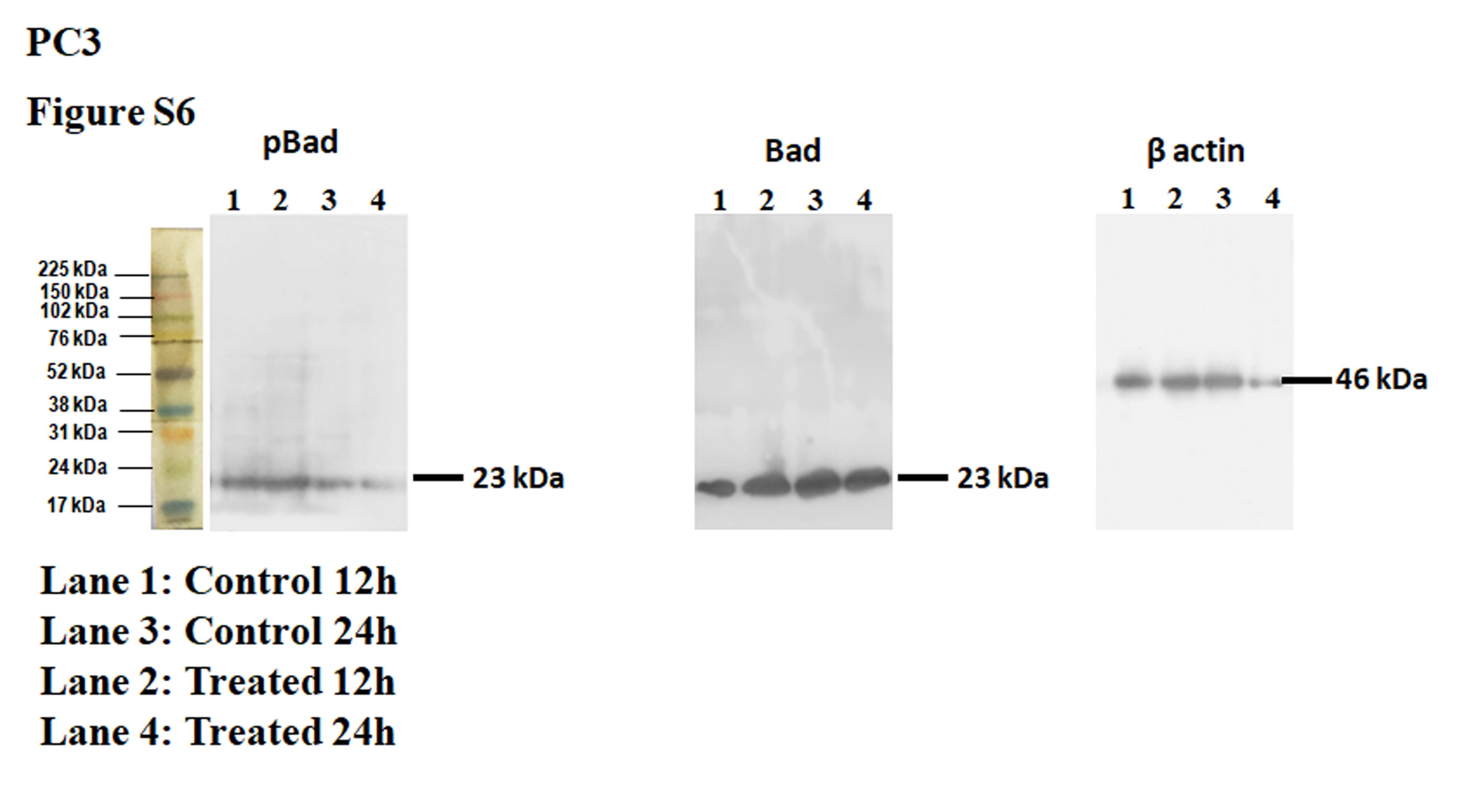

Supplement: Supplementary file 1 [file Data_Sheet_1.zip › supplementary datasheet 1/S6.tif]

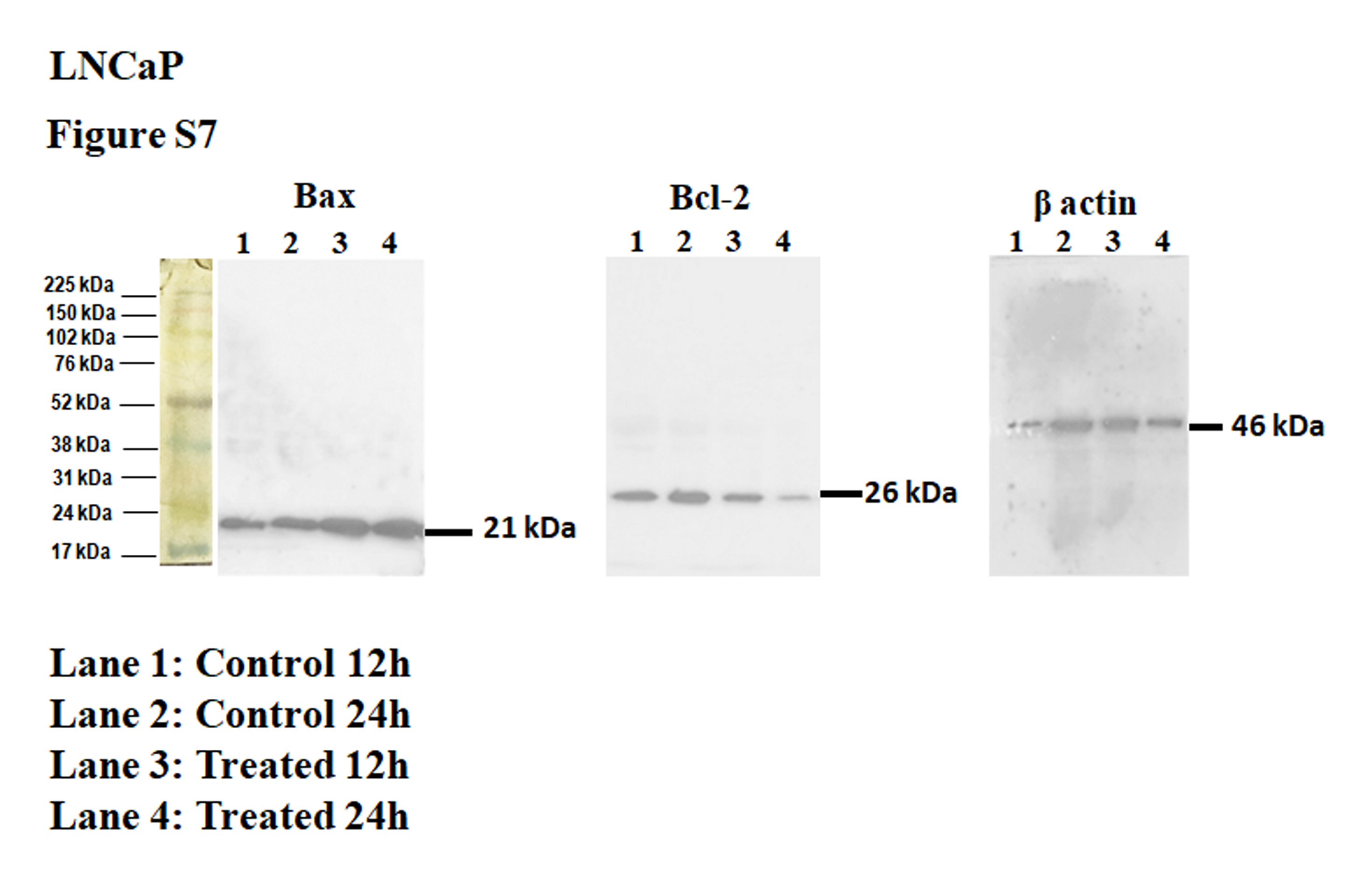

Supplement: Supplementary file 1 [file Data_Sheet_1.zip › supplementary datasheet 1/S7.tif]

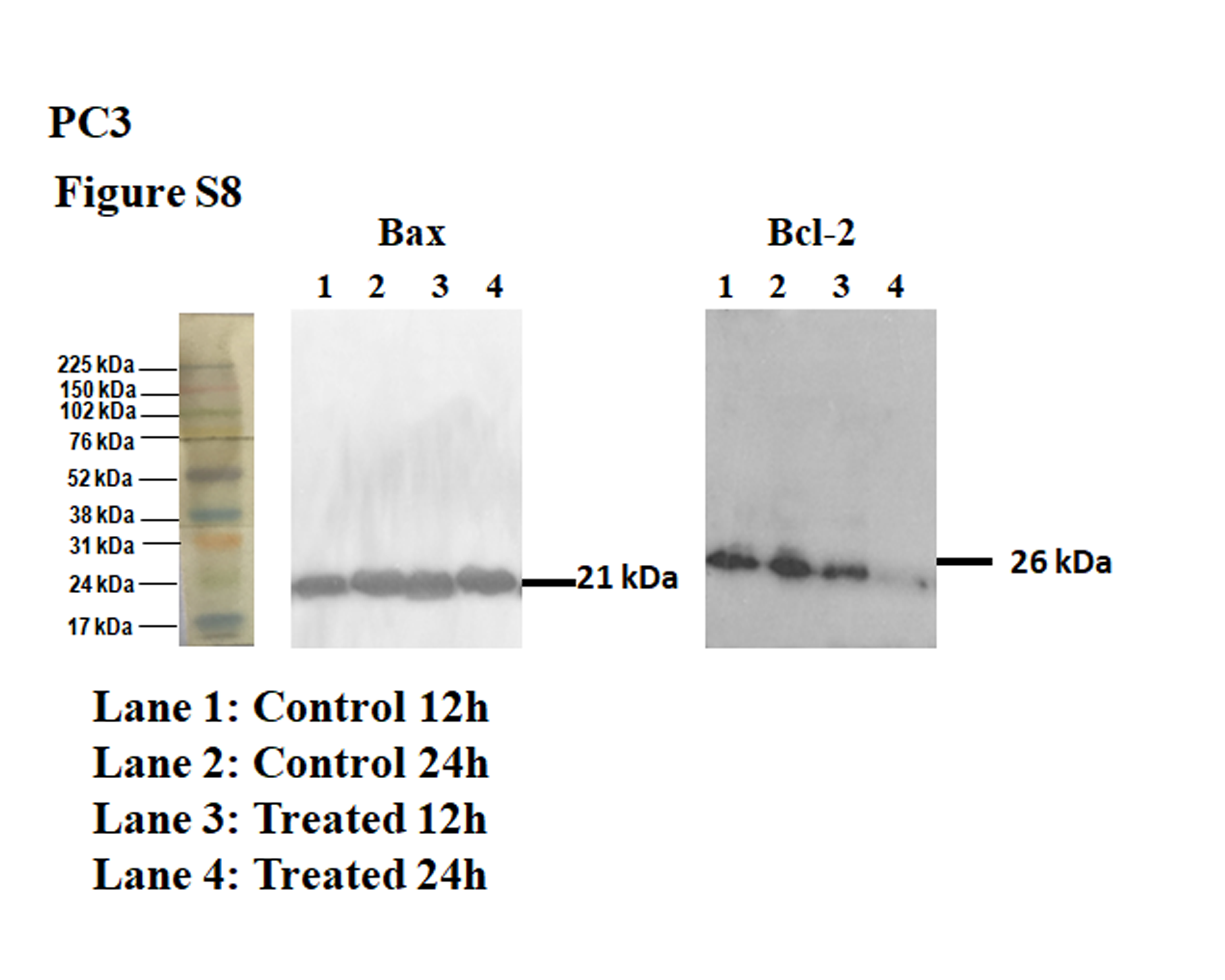

Supplement: Supplementary file 1 [file Data_Sheet_1.zip › supplementary datasheet 1/S8.tif]

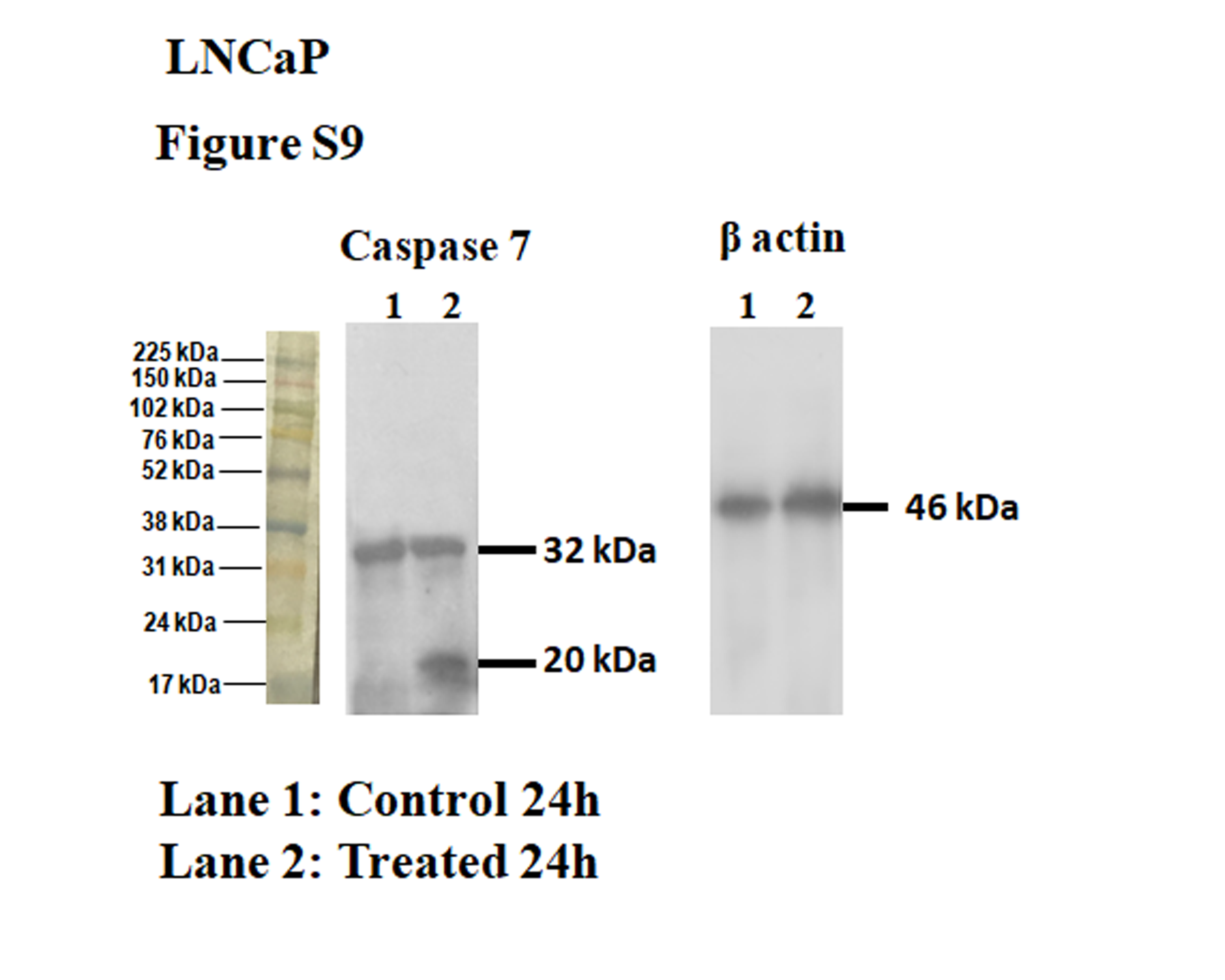

Supplement: Supplementary file 1 [file Data_Sheet_1.zip › supplementary datasheet 1/S9.tif]
